# Aberrant Functional Connectivity of Sensorimotor Network and Its Relationship With Executive Dysfunction in Bipolar Disorder Type I

**DOI:** 10.3389/fnins.2021.823550

**Published:** 2022-02-21

**Authors:** Wenjing Zhu, Wenxin Tang, Yan Liang, Xiaoying Jiang, Yi Li, Zhiyu Chen, Cheng Zhu

**Affiliations:** ^1^Hangzhou Seventh People’s Hospital, Hangzhou, China; ^2^Department of Neurology, School of Medicine, Affiliated ZhongDa Hospital, Institution of Neuropsychiatry, Southeast University, Nanjing, China

**Keywords:** bipolar disorder, executive function, resting-state fMRI, sensorimotor network, functional connectivity

## Abstract

**Background:**

The key pathophysiological mechanism of executive dysfunction in patients with bipolar disorder type I (BD-I) is still unclear. Previous studies have demonstrated that it may be related to the disbalance of the sensory motor network (SMN).

**Objective:**

This study was designed to explore the aberrant functional connectivity (FC) of SMN in BD-I patients and its potential associations with executive dysfunction.

**Methods:**

Eighteen BD-I patients and 20 healthy controls (HCs) underwent resting-state fMRI scans. The intranetwork and internetwork functional connectivities of SMN were extracted by independent component analysis (ICA). Clinical symptoms were assessed by the Bech–Rafaelsen Mania Rating Scale (BRMS) and Positive and Negative Syndrome Scale (PANSS). Executive function was measured by digit span tasks and a verbal fluency test. Finally, linear regression and correlation analyses were applied to measure the potential associations between clinical symptoms, intranetwork and internetwork functional connectivities, and executive function performance.

**Results:**

(1) Patients with BD-I showed increased connectivity in the right paracentral lobule and the right postcentral gyrus within the SMN, and the increased connectivity value was positively correlated with the BRMS score (*P* < 0.05) but negatively correlated with digit span forward scores (*P* < 0.05). (2) Compared with HC, the connectivity value increased between the SMN and dorsal attention network (DAN) (*P* < 0.01) and between the default mode network (DMN) and DAN (*P* < 0.05) but decreased between the DAN and auditory network (AN) (*P* < 0.05) and between the SMN and DMN (*P* < 0.01) in patients with BD-I. (3) Digit span forward scores and education of all participants were negatively correlated with FC between SMN and DAN. Age of all subjects was positively correlated with FC between SMN and DMN.

**Conclusion:**

Our findings suggest that the sensorimotor network of BD-I has abnormal functional connections within and between networks, and the abnormal FC value correlated with clinical symptoms and executive function, which provide new information for exploring the neural physiopathology of executive dysfunction in BD-I patients.

## Introduction

Bipolar disorder (BD) is a type of mood disorder characterized by the core feature of recurrence of mania (BD type I, BD-I) or hypomania (BD type II, BD-II) and depressive episodes with high disability and high burden (Hahn et al., 2014). Most of the findings show that BD, especially bipolar disorder type I (BD-I), is associated with deficits in cognitive functions, particularly in executive function, which correlated with the ability to integrate various skills to prepare for and execute complex behaviors (Ozonoff et al., 2004; Elshahawi et al., 2011; Liu et al., 2011; Peters et al., 2014; Drakopoulos et al., 2020). Interestingly, even modest executive dysfunction can lead to noticeable disturbance of behaviors, including deficiency in planning, organization, problem solving, and decision making, and it can persist throughout the course of the disease (Sole et al., 2016; Garcia-Laredo et al., 2021). However, the neural mechanism of executive dysfunction in patients with BD-I is still largely unclear.

Resting-state fMRI (rs-fMRI) is the spontaneous regulation of BOLD signals in the brain under the non-task state, which can reflect the process of promoting the integration of internal and external environmental neural signals. rs-fMRI is widely used in the research of a variety of mental and nervous system diseases (Dong et al., 2016; Breukelaar et al., 2020; Dimick et al., 2020; Waller et al., 2021). Many studies indicate that the brain is a complex system consisting of multiple functional networks subserving different functions, which consist of several brain regions with similar patterns of signal change over the course of rs-fMRI (Damoiseaux et al., 2006; De Luca et al., 2006; Maalouf et al., 2010; Power et al., 2011). The results of functional connectivity between and within functional networks could improve our understanding of the large-scale functional organization in executive dysfunction of patients with BD. For example, Amy Peters and his team investigated the cognitive-affective task-oriented engagement of the cognitive control network (CCN) and default mode network (DMN) may support real-world cognitive function in BD (Liu et al., 2015). Peters et al. (2020) probed the network of major depressive disorder and BD, and the results showed that memory impairment displays a central role in the cognitive impairment of patients with unipolar depression, whereas executive dysfunction appears to be more central in bipolar depression. Kristen K. Ellard investigated that impaired functional connectivity between the anterior insula and the inferior parietal lobule of the executive control network (ECN) distinguishes patients with bipolar depression from those with unipolar depression and healthy control (HC) subjects (Galimberti et al., 2020). However, there have been few studies focusing on the brain networks of patients with BD-I, and also few studies detailing how brain network abnormalities participate in the executive dysfunction of this type of patients.

An increasing number of research have shown that the DMN and sensory motor network (SMN) have a major role in emotional and cognitive processing, with the sensorimotor networks having become a focus of research on brain networks in BD in recent years (Ellard et al., 2018; Kebets et al., 2019; Magioncalda et al., 2020). The relevant research suggested that abnormality activities in SMN are the basis of emotion processing dysfunction and executive dysfunction in patients with BD. Meanwhile, the interaction between SMN and other brain networks including DMN has been shown to be dysfunctional (Martino et al., 2020). Nevertheless, how intranetwork and internetwork functional connectivities changed in the SMN of patients with BD-I, which in turn impacted human behaviors, especially executive dysfunction, has yet to be determined.

The aim of the present study is to measure the aberrant intranetwork and internetwork functional connectivities of SMN in patients with BD-I using the independent component analysis (ICA). Simultaneously, we also evaluated the patients’ clinical symptoms and executive function. Finally, we measured the potential associations between functional connectivity, clinical symptoms, and executive function in both the BD-I group and normal group. We hypothesized that the patients of BD-I have abnormal functional connections within SMN and between SMN with other networks and that the abnormal FC correlates with clinical symptoms and executive function at the same time. The functional network connectivity of SMN was involved in the executive dysfunction of BD-I patients.

## Materials and Methods

### Participants and Clinical Assessment

This study was consistent with the Declaration of Helsinki and was approved by the Hangzhou Seventh People’s Hospital Ethics Committee, and written informed consent was obtained from all participants after they had been given a complete description of the study.

Nineteen subjects with BD-I (10 males and 9 females) were recruited from the outpatient or inpatient department of the Hangzhou Seventh People’s Hospital; all subjects were medicated (with drugs including lithium carbonate, valproate, and second-generation antipsychotics). Diagnosis of BD-I was in accordance with the *Diagnostic and Statistical Manual of Mental Disorders*, fourth edition (DSM-IV). The diagnosis was conducted based on structured clinical interviews by two independent psychiatrists who had more than 10 years of clinical experience. The exclusion criteria included (1) age below 15 or above 65 years; (2) current or previous diagnosis of substance dependence, schizoaffective disorder, or schizophrenia; (3) history of brain injury, previous loss of consciousness greater than 10 min, or self-reported serious medical conditions; (4) electroconvulsive therapy history during the last 6 months; (5) any contraindications of MRI; (6) too large head motion (> 2 mm in translation or 2° in rotation). One patient was excluded because of large head motion, and the remaining 18 patients were enrolled in the research. Twenty gender-, age-, and education-matched HC volunteers (12 males and 8 females) were recruited from the community as controls *via* local advertisements. All the controls also need to accept a standard medical examination by psychiatrists to rule out the presence of current or past psychiatric illness, neurological illness, or head injury causing concussion, as well as any history of psychiatric illness in first-degree relatives. Table 1 shows the demographic and characteristic data, including neuropsychological scales, clinical characteristics, drugs of the subjects.

**TABLE 1:** Demographic and behavioral characteristic of participants.

	BD-I	HC	*^b^T*-value/χ^2^	*P*-value
Gender (male/female)^a^	10/8	12/8	0.08	0.78
Age	30.83 ± 10.28	33.30 ± 11.16	–0.71	0.49
Educational years	15.12 ± 3.22	15.56 ± 3.75	–0.51	0.67
Age of onset	22.56 ± 8.99			
Duration of illness (years)	11.45 ± 9.55			
Number of hospitalizations	5.56 ± 4.94			
Lithium (n)	8			
Anticonvulsant (n)	12			
Antipsychotic (n)	18			
Antipsychotic medication, day/mg^c,d^	605.56 ± 207.16			
BRMS	25.06 ± 6.25			
PANSS	60.11 ± 9.61			
Digit span forward	6.67 ± 1.65	8.20 ± 1.01	–3.42	0.002
Digit span backward	4.56 ± 0.78	6.90 ± 1.83	–5.22	0.001
VFT	18.72 ± 4.71	22.55 ± 5.57	–0.91	0.020
FD value	0.14 ± 0.09	0.11 ± 0.23	2.39	0.282

*BD-I, bipolar disorder type I; HC, healthy control; BRMS, Bech–Rafaelsen Mania Rating Scale; PANSS, Positive and Negative Syndrome Scale; VFT, verbal fluency test; FD, framewise displacement, used to evaluate head motion during scanning.*

*^a^Data are presented as mean ± standard deviation except gender.*

*^b^Comparisons were performed with a chi-square test for the variable of gender and independent-samples t-tests for other variables.*

*^c^All participants were taking atypical antipsychotics.*

*^d^Chlorpromazine equivalent doses were calculated.*

### Cognition and Clinical Assessment

The digit span task is a part of the executive function, which is adopted to evaluate attention (Chenji et al., 2016). All participants completed a digit span forward task followed by a digit span backward task. The digit span forward task required the participant to remember a series of numbers from two digits continuing to a maximum of 13 digits, which are presented orally. Then the participants try to verbally repeat the digits. There were two trials per digit series. All participants began with the first two digit series; if repeated correctly, they continued to the next one; otherwise, they performed the second trial at the same digit series. The task was terminated when the participant failed in the second trial. The span is defined as the maximum number of digits repeated by the participant. The digit span backward task followed the same procedure, except the order of the digits verbally repeated by the participants was reversed. During the verbal fluency test (VFT), participants were required to say as many words as possible describing an animal or a vegetable within 1 min. When the participants correctly described the animal or vegetable, 1 point was awarded. Each participant was assessed with standardized clinical instruments, including the Bech–Rafaelsen Mania Rating Scale (BRMS) and the Positive and Negative Syndrome Scale (PANSS), which were used to evaluate clinical symptoms for all patients during the 7-day period prior to the scan.

### MRI Data Acquisition

Brain structural and resting-state functional images of all subjects were acquired from the Hangzhou Seventh People’s Hospital. All participants were instructed before scanning to keep their eyes closed, relax, move as little as possible, think of nothing in particular, and not fall asleep during the scans. Resting-state MRI scans were conducted under a 1.5-T MRI scanner (Signa HDxt 1.5T, GE Healthcare, Buckinghamshire, United Kingdom) composed of 180 echo-planar imaging volumes with the following parameters: TR = 2,000 ms; TE = 40 ms; flip angle = 85°; matrix size = 64 × 64; field of view = 240 × 240 mm; slice thickness = 3 mm; and 28 continuous slices. The acquisition time is 6 min. A T1-weighted structural image was also acquired for each patient to further elucidate and discard gross radiological alterations (TR = 9.5 ms; TE = 3.1 ms; flip angle = 20°; field of view = 240 mm × 240 mm; slice thickness = 1.2 mm).

### Functional MRI Data Processing

Functional MRI data were preprocessed using the Statistical Parametric Mapping software (SPM12)^1^ and Data Processing and Analysis for Brain Imaging (DPABI)^2^ (Groth-Marnat and Baker, 2003). The data preprocessing includes the following steps:

(1) Discarding of the first five volumes to achieve equilibrium and a steady state.
(2) Slice timing correction.
(3) Realignment (head motion parameters were computed by estimating the translation in each direction and the angular rotation on each axis for each volume; we required the translational or rotational motion parameters to be less than 2 mm or 2°; the framewise displacement (FD), which indexes the volume-to-volume changes in head position was also calculated).
(4) These images were then spatially normalized by the following steps: individual structural images were co-registered with the mean functional image, firstly. Then the transformed structural images were segmented and normalized to the Montreal Neurological Institute (MNI) space using a high-level non-linear warping algorithm which uses the exponentiated Lie algebra (DARTEL) technique to acquire the diffeomorphic anatomical registration (Yan et al., 2016). Finally, each functional volume was spatially normalized to the MNI space using the deformation parameters estimated during the above step and resampled into a 3-mm cubic voxel.
(5) Spatial smoothing with a Gaussian kernel of 6 mm × 6 mm × 6 mm.

### Independent Component Analysis

This research applied the group ICA tool named GIFT (version 3.0b)^3^ to perform group spatial ICA. We analyzed a mean of 16 components for each subject by the minimum description length criteria. Spatial ICA decomposes the participant data into linear mixtures of spatially independent components that exhibit a unique time course profile. Two data reduction steps can achieve this goal: Firstly, applying principal component analysis to reduce the subject-specific data into 24 principal components; and secondly, concatenating reduced data of all participants across time and using the infomax algorithm to decompose the data into 16 independent components. To ensure estimation stability, the infomax algorithm was repeated 20 times in ICASSO,^4^ and the most central run was selected and analyzed further. Finally, the GICA back-reconstruction approach was used to find the individual time courses and spatial maps of the participants.

**FIGURE 1:**
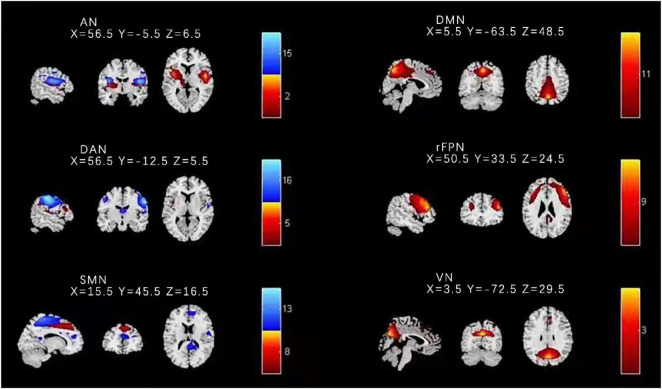
Spatial distribution of the nine independent components for all HC and BD-I subjects. Components 8 and 13 were found to match with the SMN. DMN, default mode network; rFPN, right frontoparietal network; DAN, dorsal attention network; SMN, sensorimotor network; AN, auditory network; VN, visual network.

We applied several independent components that had peak activations in gray matter; showed low spatial overlap with heartbeat, motion, and susceptibility artifacts; and exhibited low frequency power primarily to identified functional networks; then we used the methods of visual inspection and spatial network template to acquire the target brain network. In the step of visual inspection, the SMN includes the somatosensory and motor cortices (Ashburner, 2007). Finally, this research procedure resulted in six functional networks out of the 16 independent components obtained (Figure 1): DMN, right frontoparietal network (rFPN), dorsal attention network (DAN), SMN, auditory network (AN), and visual network (VN).

The following were performed in the intranetwork functional connectivity calculation. First, a one-sample *t*-test was carried out to determine anatomical regions within SMN at a threshold of *P* < 0.05, FEW corrected. Second, a two-sample *t*-test was performed for SMN masked by the regions identified in one-sample *t*-tests with age and gender as a nuisance covariate to identify the significantly different regions between HC and BD subjects (FEW-corrected *P* < 0.001).

In the internetwork functional connectivity calculation, the value of function connectivity was estimated by Pearson correlation coefficients between pairs of time courses of the functional networks, resulting in a symmetric 9 × 9 correlation matrix for each participant. Finally, we used Fisher’s transformation to transform correlations to *z*-scores, which can improve the normality. Intranetwork connectivity was examined *via* the spatial maps, indexing the contribution of the time course to each voxel comprising a given component.

### Statistical Analysis

The statistical descriptive analyses of demographic and behavioral data were conducted using the SPSS 17.0 software package. We performed multiple regression analyses to investigate the relationships between intranetwork and internetwork functional connectivity of SMN with age, education years, and behavioral performance (e.g., BRMS, PANSS, and cognitive function). Significance was determined by *p* < 0.05 (two-tailed), with no correction.

## Results

### Demographic and Behavioral Performance

Table 1 describes the demographic, behavioral performance, and head motion data of 18 patients with BD-I and 20 HCs. There was no significant difference between two groups in terms of age, gender, and head motion. Compared with the HCs, patients with BD-I presented greater scores in BRMS and PANSS and lower scores in digit span and VFT.

### Connectivity Within Sensory Motor Network

Based on previous studies and the highest ranked correlation values calculated by the spatial network template in this study, components 8 and 13 were found to match with the SMN. The other seven components were separately identified as the DMN, rFPN, DAN, AN, and VN. Figure 1 shows the results of identified spatial network maps.

**FIGURE 2:**
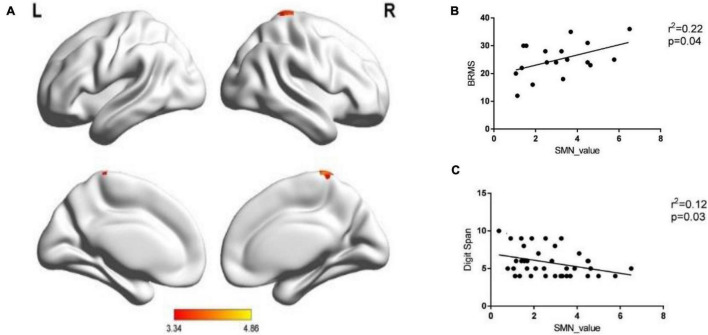
The within-network connectivity of SMN. **(A)** Patients BD-I showed increased within-network connectivity in the right paracentral lobule and the right postcentral gyrus in the SMN compared with HCs. **(B)** The correlation between the within-network connectivity value of SMN in patients and the BRMS score. **(C)** The correlation between the within-network connectivity value of SMN in all subjects and digit span forward scores.

A two-sample *t*-test was performed when analyzing the within-network connectivity for SMN, and we found the brain areas with significant differences in the network between the two groups. Significantly increased within-network connectivity was found in the right paracentral lobule (voxel size = 26; peak coordinate = 8, −32, 68; *T* = 4.86, *P* < 0.001) and the right postcentral gyrus (voxel size = 20; peak coordinate = 41, −26, 53; *T* = 3.33, *P* < 0.001) in the SMN of the BD-I group (Figure 2A), and there were no clusters of reduced connectivity in BD-I patients compared to HCs.

The increased within-network connectivity value in the SMN was positively correlated with the BRMS score (*P* < 0.05) and negatively correlated with the digit span forward score (*P* < 0.05). There was no significant correlation in the rest (all *P* > 0.05, see Figures 2B,C).

### Connectivity Between Sensory Motor Network and Other Networks

**FIGURE 3:**
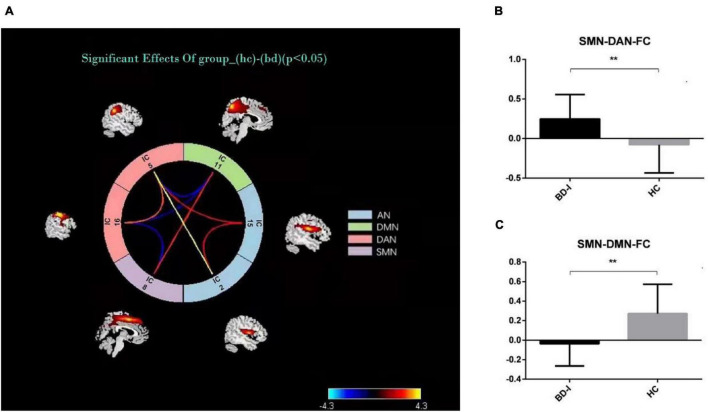
**(A)** Internetwork functional connectivity in BD-I and HCs. **(B)** Comparison of functional connectivity between SMN and DAN in the two groups. **(C)** Comparison of functional connectivity between SMN and DMN in the two groups. *p < 0.05; **p < 0.01.

**FIGURE 4:**
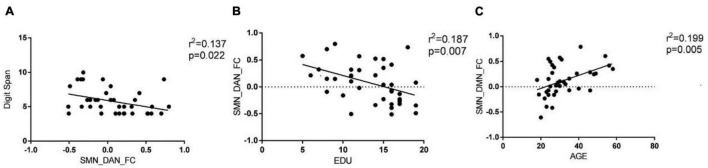
Pearson correlation analysis of internetwork functional connectivity value and clinical information. **(A)** Digit span forward scores of all participants were negatively correlated with FC between SMN and DAN (*P* < 0.05). **(B)** Education years of all participants were negatively correlated with FC between SMN and DAN (*P* < 0.05). **(C)** Age of all subjects was positively correlated with FC between SMN and DMN (*P* < 0.05).

The results of connectivity differences between networks in BD-I patients vs. HCs are shown in Figure 3A. Both positive and negative internetwork functional connectivities were observed. As observed from Figures 3B,C, compared with those in HCs, the functional connectivity value increased between SMN and DAN and decreased between SMN and DMN in BD-I subjects (*P* < 0.01, FDR corrected). Moreover, as observed from Figures 4A–C, digit span forward scores and education years of all participants were negatively correlated with FC between SMN and DAN (*P* < 0.05). The age of all subjects was positively correlated with FC between SMN and DMN (*P* < 0.05).

## Discussion

### Main Findings

Our main findings are as follows. (1) compared with HC, patients with BD-I showed increased within-network connectivity in the right paracentral lobule and the right postcentral gyrus in the SMN, and the connectivity value was positively correlated with BRMS score but negatively correlated with digit span forward scores. (2) The between-network connectivity of BD-I increased between SMN and DAN and between DMN and DAN but decreased between DAN and AN and between SMN and DMN when compared to HC. (3) Digit span forward scores and education of all participants were negatively correlated with FC between SMN and DAN. Age of all subjects was positively correlated with FC between SMN and DMN. Aberrant intranetwork and internetwork functional connectivities of sensorimotor network may be the neural mechanism of interaction between clinical symptoms and executive dysfunction in patients of BD-I.

### Executive Dysfunction of Bipolar Disorder Type-I

The two main impaired elements in individuals with BD are emotional processing and cognitive functions (Wessa and Linke, 2009; Wang C. et al., 2020). Failing in using cognitive functions to regulate and maintain emotional states can lead to a dysfunction in their emotional processing and emotion regulation (Torrent et al., 2006). The present study applied the digit span tasks and VFT to investigate executive function. The result shows that valuations of digit span tasks and VFT of BD-I patients were lower than those of HCs at baseline, and it is consistent with previous related researches (Phillips and Vieta, 2007; Kosger et al., 2015). However, the digit span tasks and VFT are just parts of the executive function, and we need more research with multiple tests to support our results.

### Intranetwork Connectivity in Bipolar Disorder Type-I

Our research found increased within-network connectivity in the right paracentral lobule and the right postcentral gyrus in the SMN, and the connectivity value was positively correlated with the BRMS score. The results agreed with those of Erol et al. (2013) and Khadka et al. (2013). Kahdka reported that BD-I patients with psychosis episodes showed increased FC in the superior frontal gyrus and medial frontal gyrus in the SMN compared with HCs. Ishida et al. (2017) and Kazemi et al. (2018) found within-network connectivity in the right premotor region in the SMN was positively correlated with the total score of YMRS for BD subjects. As known from previous studies, the SMN mainly regulates sensory and motor functions, and the abnormal connection of this network function corresponds well to the patients’ clinical symptoms. The anterior part of the paracentral lobule is located in the primary motor cortex, which regulates voluntary movement of the body. The concentration of norepinephrine in patients with BD-I is increased, accompanied by abnormalities of dopamine (DA) and γ-aminobutyric acid (GABA), which leads to the increased function of the primary motor cortex. The central posterior gyrus belongs to the primary sensory cortex and receives various sensory stimuli. The patients’ right central posterior gyrus functional connection is abnormal, which may be related to the patients’ increased motor function and compensation of adjacent sensory cortex. More importantly, different from previous SMN studies, we found that the increased connectivity of SMN was negatively correlated with digit span forward scores. In the pre-language stage of individual development, the occurrence of cognition mainly depends on sense and kinesthesia. Even if the individual gradually relies on language for learning and communication with development, sensorimotor ability is still a basic ability for the individual to recognize external things (Supekar et al., 2019). Furthermore, patients with BD-I often suffer from the inability of the brain to filter and screen irrelevant information, and the inhibitory function is impaired, leading to excessive information flooding into the consciousness and then various abnormal sensory experiences, which ultimately affect cognitive function. All these researches show that the SMN plays an important role in emotional dis-regulation and cognitive dysfunction of BD-I patients.

### Internetwork Connectivity in Bipolar Disorder Type-I

The results of the second part of this study show that the between-network connectivity value of BD-I increased between SMN and DAN but decreased between SMN and DMN when compared to HC. The brain is a complex brain network, which is composed of multiple functionally interacting sub-networks (Mochizuki-Kawai et al., 2004). Previous studies have shown that the SMN is the core network that is vulnerable to dysfunction in emotional functions, emotion recognition, and cognitive functions of BD (Wood et al., 2016; Wang J. et al., 2020). So advanced CCNs related to sensorimotor functions, such as the DMN, ECN, and DAN, may also experience functional impairment or compensation, the so-called functional reorganization (Davis et al., 2017). Wang et al. (2014) found that the functions of the medial prefrontal lobe and the cingulate gyrus of DMN are related to the planning, preparation, and execution of exercise. Damage to this area will affect the intentional purpose and rough planning of the motor cortex during exercise. Roelcke et al. (1997) revealed that an imbalance was observed in the DMN/SMN activity of bipolar patients, and a low ratio of DMN/SMN activity was reported in the manic phase while the opposite happened in the depression phase. Our results are consistent with these conclusions and also show that age was positively correlated with FC between SMN and DMN, which suggests that different age groups of BD-I patients have different degrees of imbalance in DMN/SMN (Fox et al., 2006; Chen et al., 2020; Manza et al., 2020). The relationship between DMN and SMN was considered as a diagnostic marker for BD. The DAN mainly includes the bilateral parietal internal sulcus and the joint cortical area of the superior frontal gyrus and the central anterior gyrus. It is related to the active attention process in the task and is obviously activated in tasks where attention cues such as target, position, and time appear. The upper leaflet of DAN is responsible for receiving the input of visual information and plays a role of visual control in motor tasks. Patients with BD-I have the characteristics of increased active attention and shifting attention with the environment. The functional connection between DAN and SMN is enhanced, and intriguingly, it is negatively correlated with digit span forward scores and education years of all participants, which may be consistent with the clinical features of increased volitional activity in patients with BD-I and also suggests that high years of education may be a protective factor for the disease. The results of the study confirm that the patients’ proprioceptive perception and visual information are impaired by the top-down and bottom-up two-way adjustment and control effects during the completion of motor coordination, which also affects the patients’ executive function accordingly. The two-way regulation and control function of DAN/SMN gives us a new understanding of the imbalance between DAN and SMN networks.

## Conclusion

In conclusion, we got valuable information about aberrant intranetwork and internetwork functional connectivities of SMN in patients with BD-I, compared with HCs. Furthermore, we found a significant relationship between the abnormal intranetwork and internetwork functional connectivity values and clinical symptoms and executive function, which provides new information for exploring the neural physiopathology of executive dysfunction in BD-I patients.

### Limitations

Some limitations in this study should be addressed. First, our study enrolled a small-sized sample. Larger samples in the future are needed to confirm current findings. Second, all patients included in this study were administrated with drugs, which made the interpretation of results complex and difficult, but it is also a high risk in the image acquisition of untreated patients with manic episodes. After the sample size is expanded, interfering factors such as the duration of illness, the number of years of therapy, and the number of hospitalizations can be analyzed by comparing patient groups. Third, this study only included BD-I patients during manic episodes but not depressive episodes, which will make the attribution of executive dysfunction in BD-I patients incomplete.

## Data Availability Statement

The original contributions presented in the study are included in the article/supplementary material, further inquiries can be directed to the corresponding author/s.

## Ethics Statement

The studies involving human participants were reviewed and approved by the Hangzhou Seventh People’s Hospital. Written informed consent to participate in this study was provided by the participants’ legal guardian/next of kin.

## Author Contributions

CZ and ZC supervised the present study. WZ and WT performed the analysis and wrote the manuscript. YaL, XJ, and YiL helped to collect the data. All authors contributed to the article and approved the submitted version.

## Conflict of Interest

The authors declare that the research was conducted in the absence of any commercial or financial relationships that could be construed as a potential conflict of interest.

## Publisher’s Note

All claims expressed in this article are solely those of the authors and do not necessarily represent those of their affiliated organizations, or those of the publisher, the editors and the reviewers. Any product that may be evaluated in this article, or claim that may be made by its manufacturer, is not guaranteed or endorsed by the publisher.

## Footnotes

1
http://www.fil.ion.ucl.ac.uk/spm

2
http://rfmri.org/dpabi

3
mialab.mrn.org/software/gift/

4
http://research.ics.tkk.fi/ica/icasso/
