# Corrigendum: Aberrant functional connectivity of sensorimotor network and its relationship with executive dysfunction in bipolar disorder type I

**DOI:** 10.3389/fnins.2024.1515904

**Published:** 2024-11-05

**Authors:** Wenjing Zhu, Wenxin Tang, Yan Liang, Xiaoying Jiang, Yi Li, Zhiyu Chen, Cheng Zhu

**Affiliations:** ^1^Hangzhou Seventh People's Hospital, Hangzhou, China; ^2^Department of Neurology, School of Medicine, Affiliated ZhongDa Hospital, Institution of Neuropsychiatry, Southeast University, Nanjing, China

**Keywords:** bipolar disorder, executive function, resting-state fMRI, sensorimotor network, functional connectivity

In the published article, there was an error in the Funding statement. The funding statement for the Youth Innovative Talent Support Program Fund of Zhejiang Provincial Department of Health was displayed as “2022493976” instead of “2022RC061”. The correct Funding statement appears below.

